# Pursuing an Optimal Regional Analgesia Strategy for Total Knee Arthroplasty: A Double-Blind Randomized Controlled Study of Femoral Triangle, Adductor Canal, and Dual Subsartorial Blocks (FAD Trial)

**DOI:** 10.7759/cureus.91147

**Published:** 2025-08-27

**Authors:** Kartik Sonawane, Shlok Saxena, Tuhin Mistry, Jagannathan Balavenkatasubramanian, Dhanasekaran Soundarrajan, Shanmuganathan Rajasekaran

**Affiliations:** 1 Anesthesiology, Ganga Medical Centre and Hospitals, Pvt. Ltd, Coimbatore, IND; 2 Orthopedics and Joint Replacement, Ganga Medical Centre and Hospitals, Pvt. Ltd, Coimbatore, IND; 3 Orthopedics and Spine Surgery, Ganga Medical Centre and Hospitals, Pvt. Ltd, Coimbatore, IND

**Keywords:** adductor canal block, dual subsartorial block, fad trial, femoral triangle block, motor-sparing analgesia, opioid-free recovery, regional anesthesia, total knee arthroplasty

## Abstract

Background

Total knee arthroplasty (TKA) is often associated with intense postoperative pain, which can delay mobilization and hinder recovery. While motor-sparing blocks such as the femoral triangle block (FTB) and adductor canal block (ACB) are commonly used, both offer incomplete analgesic coverage. To overcome these limitations, the dual subsartorial block (DSB) was introduced as a procedure-specific, motor-sparing technique that combines and modifies FTB and ACB into a dual-injection approach for enhanced efficacy.

Objective

The objective of this study is to compare the analgesic efficacy, motor-sparing effect, opioid-sparing potential, and patient satisfaction among three regional analgesia (RA) techniques, FTB, ACB, and DSB, in patients undergoing TKA.

Methods

This prospective, double-blind, monocentric trial enrolled 120 patients undergoing unilateral primary TKA, randomized equally into FTB, ACB, or DSB groups (n = 40 each). All patients received standardized spinal anesthesia followed by the assigned ultrasound-guided block. The primary outcome was postoperative quadriceps strength. Secondary outcomes included visual analog scale (VAS) pain scores, rescue opioid use, pain location mapping, patient satisfaction, and block duration.

Results

Quadriceps strength was preserved in all groups. DSB showed significantly better static and dynamic pain control (p < 0.001), with zero opioid use compared to minimal use in FTB and highest use in ACB. Pain mapping revealed incomplete coverage with ACB, particularly at upper incision sites. DSB provided the longest block duration (>24 hours) and the highest satisfaction scores.

Conclusions

DSB, which strategically integrates and modifies FTB and ACB, offers superior, comprehensive, and motor-sparing analgesia. Its enhanced anatomical precision and functional benefits suggest that DSB can be considered a reliable, procedure-specific RA technique with a strong potential to improve outcomes in modern TKA care pathways.

## Introduction

Osteoarthritis (OA) is the leading cause of disability among older adults [1]. About 9.6% of men and 18% of women over 60 have symptomatic OA [2]. Eighty percent report mobility limitations, and 25% struggle with daily activities [3]. Chronic pain and immobility reduce quality of life and contribute to psychological distress and increased mortality risk [4-7]. Total knee arthroplasty (TKA) is the standard treatment for end-stage OA, restoring function and improving quality of life [8-9]. However, it is associated with intense postoperative pain. Inadequate analgesia may delay recovery and cause persistent pain in up to 40% of patients [10-12]. Effective perioperative pain control is essential for pain relief, opioid minimization, and early mobilization, as emphasized in enhanced recovery after surgery (ERAS) protocols [13-15].

While femoral nerve block (FNB) was historically the mainstay, its association with quadriceps weakness and fall risk led to the emergence of motor-sparing alternatives such as the adductor canal block (ACB) and femoral triangle block (FTB) [16,17]. These techniques preserve strength but often fail to provide complete sensory coverage, requiring additional interventions [18,19]. To address these gaps, our institution developed the dual subsartorial block (DSB) in 2017, integrating modified versions of ACB and FTB to enhance coverage while preserving motor function [20-25]. Its consistent clinical success prompted the need for formal evaluation to justify its broader adoption.

In this study, we aim to critically assess both components of DSB: FTB and ACB to validate the technique’s adaptability and long-term utility. This first-of-its-kind direct comparison seeks to validate DSB’s analgesic performance and confirm its non-inferior motor preservation, thereby reinforcing its adoption as an optimal regional analgesia (RA) strategy for TKA. We focus primarily on quadriceps strength as a key indicator of motor-sparing efficacy. We hypothesized that DSB would preserve quadriceps strength comparably to ACB and FTB, while offering superior analgesic benefits.

## Materials and methods

Study design and setting

This was a prospective, double-blind, randomized controlled trial (RCT) conducted at a tertiary care center between November 2021 and December 2022 (first enrollment: 25/11/2021). It was approved by the Institutional Ethics Committee and prospectively registered in the Clinical Trials Registry of India (CTRI/2021/11/038194; dated 23/11/2021; available at: https://ctri.nic.in/Clinicaltrials/pmaindet2.php?EncHid=NjA1Nzc=&Enc=&userName=).

Participants/study subjects

Eligible patients were aged 18-75 years, ASA (American Society of Anesthesiologists) grade I or II, undergoing unilateral cemented TKA via the medial approach under neuraxial anesthesia by a single surgeon. Exclusion criteria included severe deformities, comorbidities, or contraindications as summarized in Table 1.

**Table 1:** Inclusion and Exclusion Criteria. ASA, American Society of Anesthesiologists; TKA, total knee arthroplasty.

Inclusion Criteria	Exclusion Criteria
Age 18–75 years	ASA III or IV
ASA grade I or II	Severe valgus deformity
Elective unilateral cemented TKA	Advanced hepatic/renal disease
Medial approaches by a single surgeon	Cognitive/neurological dysfunction
	Contraindications to neuraxial block or study drugs
	Coagulopathy
	Chronic opioid use (>3 months)
	Operative limb neuropathy
	Revision/augmented/bilateral TKA
	Refusal to participate

Sample size estimation

This study was designed as a three-arm, parallel-group RCT with a 1:1:1 allocation ratio (FTB, ACB, DSB). The primary endpoint was postoperative quadriceps strength measured on the 0-5 MRC scale. Our pilot study on DSB served as the basis for determining the final participant count [20]. The following formula was used to determine the number of samples required for each arm of the study.

n = [2 × (Zα/2 + Z1-β)² × σ²] / δ²​​

Where Zα/2 is the critical value for a two-sided test at a significance level (α) of 5%, thus Zα/2 = 1.96, and with a Type II error (β) of 10%, yielding a power (1-β) of 90% and therefore Z1-β = 1.28. Based on an expected mean difference (δ) of 0.81 and a pooled standard deviation (σ) of 1.12 from the pilot study, the required sample size was ≈40.14 per group, rounded up to 41. 

n = [2 × (1.96 + 1.28)² × (1.12)²] / (0.81)² ≈ 40.14 =41

Accounting for a ~5% attrition rate, the target enrollment was set at 120 participants (40 per group). No attrition occurred, so the final analyzed sample equaled the target sample size. Thus, a total of 120 patients were enrolled in the study.

Pairwise comparisons (DSB vs. FTB and DSB vs. ACB) were analyzed with a Bonferroni adjustment to account for multiplicity. Although the MRC scale is ordinal, prior studies and our pilot data showed near-normal distribution of scores, supporting its use as a continuous variable for sample size estimation and analysis.

Randomization and blinding

Following Institutional Review Board approval and written informed consent, 120 patients were randomly assigned (using the computer-generated method) to three equal groups (40 each) of patients: FTB, ACB, and DSB. Apart from the researchers who performed the blocks, all other investigators, including anesthesiologists, surgeons, physiotherapists, nurses, and study subjects, were blinded to the randomization.

Patient preparation

Preoperatively, after obtaining intravenous access, patients received approximately 10 mL/kg of Ringer’s lactate solution tailored to their hemodynamic status. During the operation, routine anesthetic monitoring was performed using non-invasive blood pressure, pulse oximetry, and electrocardiography. All patients underwent spinal anesthesia administered in the standard manner (L3-L4) with 10-15 mg of hyperbaric bupivacaine 0.5%. Additionally, all patients were sedated with 0.01 mg/kg midazolam as needed.

Immediately after surgery and dressing application, an experienced anesthesiologist (with over five years of experience in ultrasound-guided RA techniques) performed ultrasound-guided FTB/ACB/DSB as allocated using a prepared local anesthetic (LA) solution (0.2% ropivacaine + 8 mg dexamethasone). Postoperatively, all patients were monitored in the high-dependency unit for 24 hours for postoperative care and pain management. Patients continued on the hospital’s standard multimodal analgesia (MMA) protocol (Table 2) throughout their stay.

**Table 2:** Perioperative Pharmacological Regimen. IV, intravenous; IM, intramuscular.

Time Point	Medication Regimen
Night before surgery	Oral Paracetamol 1 gm
	Pantoprazole 40 mg
	Pregabalin 75 mg
	Aceclofenac 100 mg
One hour before surgery	IV Ramosetron 0.3 mg
	IV Tranexamic Acid 1 gm
	IV Paracetamol 1 gm
Intraoperative (before incision)	IV Ketorolac 30 mg
	IV Dexamethasone 8 mg
	Adequate IV maintenance fluids
Postoperative (first 24 hours)	IV Paracetamol 1 gm (every six hours)
	IV Ketorolac 30 mg (every 12 hours)
	IV Pantoprazole 40 mg (once daily)
	Transdermal Buprenorphine patch 5–10 mg (for seven days)
	IM Butadol 1 mg + Phenergan 12.5 mg (night sedation)
Postoperative (after 24 hours)	Oral Paracetamol 1 gm (four times daily)
	Pantoprazole 40 mg (once daily)
	Pregabalin 75 mg (nightly)
	Aceclofenac 100 mg (twice daily)
	Ecosprin 150 mg (once daily)

Block interventions

**Figure 1:**
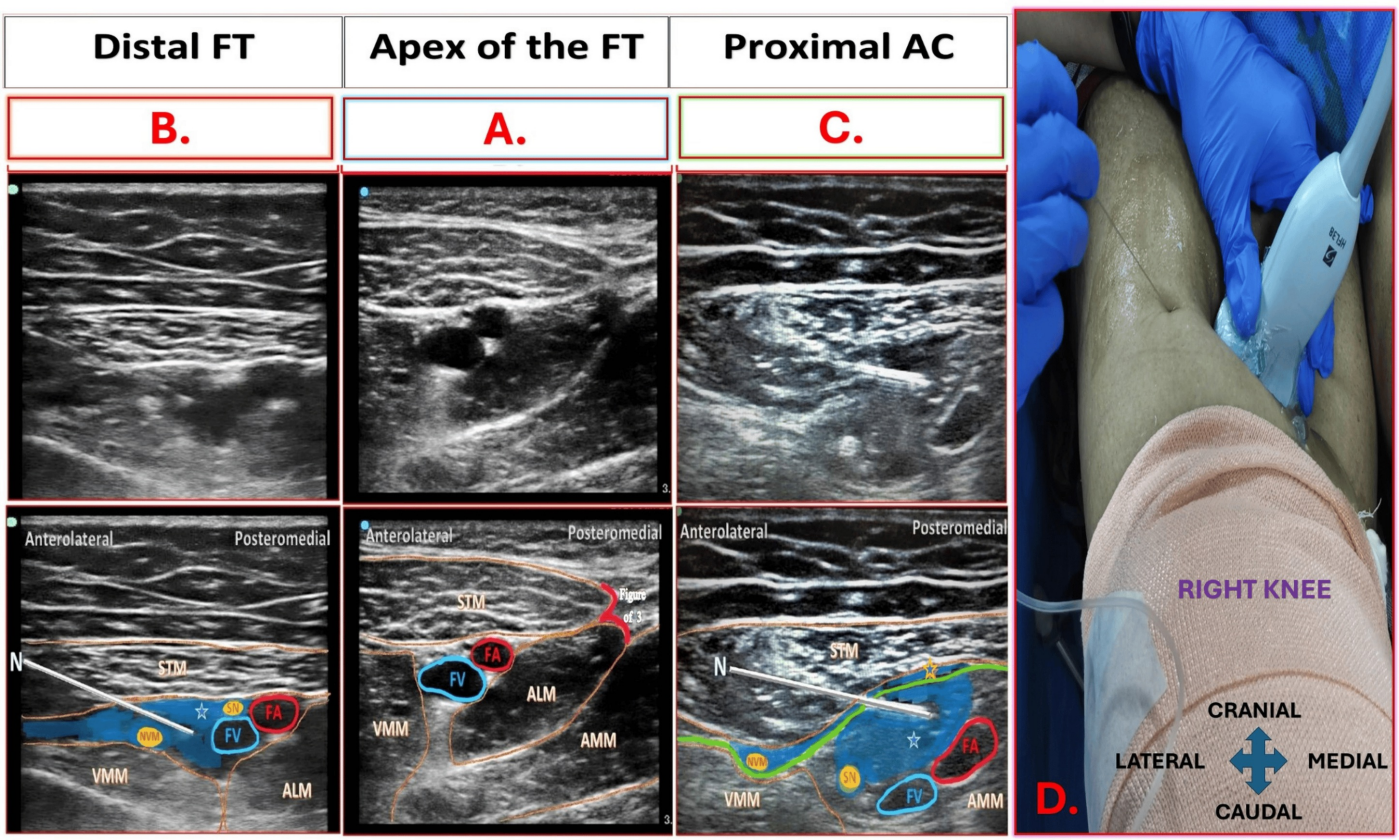
Ultrasound Probe Placement and Sonoanatomy of the Dual Subsartorial Block (DSB). The figure illustrates probe positioning, needle approach, and sonoanatomy for the DSB, comprising A. Apex of the Femoral Triangle: Identified at the intersection of the adductor longus (ALM) and sartorius (STM) muscles, forming the “figure-of-3” or “kissing sign.” B. Distal Femoral Triangle Block: Performed 1–2 cm proximal to the apex. C. Proximal Adductor Canal Block: Performed 1–2 cm distal to the apex. D. Ultrasound Probe Position and Needle Direction: Probe placed medially on the thigh with an in-plane needle approach shown. Accurate identification of the apex (A) is crucial. The DSB is performed sequentially: first identifying the apex (A), then injecting proximally (B) and distally (C) for optimal analgesic spread. STM, sartorius muscle; ALM, adductor longus muscle; VMM, vastus medialis muscle; AMM, adductor magnus muscle; FA, femoral artery; FV, femoral vein; SN, saphenous nerve; NVM, nerve to vastus medialis; Blue area, local anesthetic drug spread; Green line, vastoadductor membrane; Blue star with blue border, drug spread below VAM in adductor canal; Blue star with orange border, drug spread above VAM. Source: Adapted from Sonawane et al. under CC BY-NC 4.0 [22].

This study compared the DSB with its components, FTB and ACB (Table 3). The first step of all three techniques included identifying the apex of the femoral triangle (FT) formed by the intersection of the medial borders of the sartorius muscle (STM) and adductor longus muscle (ALM), appearing as a “sign of 3” or “kissing sign,” under ultrasound (Figure 1A).

**Table 3:** Comparative Characteristics of FTB, ACB, and DSB Techniques. FTB, femoral triangle block; ACB, adductor canal block; DSB, dual subsartorial block; FT, femoral triangle; STM, sartorius muscle; VMM, vastus medialis muscle; AC, adductor canal; VAM, vastoadductor membrane; FA, femoral artery; LA, local anesthetic; NVM, nerve to vastus medialis; SN, saphenous nerve; ERAS, enhanced recovery after surgery.

Parameter	FTB	ACB	DSB
Sonoanatomical landmark	Apex of FT (sign of 3')	Apex of FT (sign of 3')	Apex of FT (sign of 3')
Site of injection	1–2 cm proximal to apex, between STM and VMM (not in true distal FT)	1–2 cm distal to apex in proximal AC, below VAM, adjacent to superficial FA	Sequential injections: 1–2 cm proximal + 1–2 cm distal to apex
LA solution	20 ml of 0.2% ropivacaine + 8 mg dexamethasone	20 ml of 0.2% ropivacaine + 8 mg dexamethasone	40 ml of 0.2% ropivacaine + 8 mg dexamethasone
LA volume	20 ml	20 ml	20 ml + 20 ml (dual injections)
Targets	NVM and SN (STM–VMM plane)	SN, perivascular space below VAM	First: NVM and SN (STM–VMM); Second: SN, perivascular space below VAM
Coverage	Extra-articular pain generators	Intra-articular > partial extra-articular	Extra-articular, intra-articular, and posterior knee
Procedure-specific	No	No	Yes
Motor-sparing	Yes	Yes	Yes
Opioid-sparing	Better	No	Best
Precision injection	Yes (modified technique)	Yes (modified technique)	Yes (modified technique)
ERAS suitability	Yes	Yes	Yes

The FTB technique included administration of a single injection of 20mL LA solution 1-2 cm proximal to the apex of the FT, targeting the saphenous nerve (SN) and the nerve to vastus medialis (NVM), separating the intermuscular plane between STM and vastus medialis muscle (VMM) (Figure 1B). The ACB technique included administration of a single injection of 20 mL LA solution 1-2 cm distal to the apex of the FT (in the proximal AC), targeting SN located under the vastoadductor membrane (VAM) and adjacent to the femoral artery (FA) (Figure 1C). The DSB technique included a dual-injection approach combining and modifying conventional FTB and ACB (Table 4). The first injection of 20 mL LA solution (distal FTB) was administered 1-2 cm proximal to the apex of the FT, targeting the SN and NVM in the plane between the STM and VMM, thereby separating this intermuscular plane. The second injection (proximal ACB) of 20 mL of LA solution was administered 1-2 cm distal to the apex of the FT, under the VAM, beside the FA, while visualizing the disappearing LA spread due to FA compression. All three techniques differ in their approach and extent of blockade, with DSB representing a refined combination of FTB and ACB. The ultrasound probe position transversely over the medial thigh, and the corresponding needle direction for all interventions is depicted in Figure 1D. The apex of the FT serves as a critical sonoanatomical landmark, consistently guiding accurate LA deposition into the intended territories during block administration [21-23].

**Table 4:** Modifications in DSB Compared to Conventional FTB and ACB. FTB, femoral triangle block; ACB, adductor canal block; DSB, dual subsartorial block; FT, femoral triangle; STM, sartorius muscle; VMM, vastus medialis muscle; AC, adductor canal; VAM, vastoadductor membrane; FA, femoral artery; LA, local anesthetic; NVM, nerve to vastus medialis; SN, saphenous nerve; TKA, total knee arthroplasty.

Parameter	Conventional FTB	Conventional ACB	DSB
Block type	Single-injection	Single-injection	Dual injections below the STM
Anatomical landmark	Variable (not fixed)	Mid-thigh level	Apex of FT (sign of 3) for both injections
Injection site	Within the distal FT	Mid-thigh AC	FTB: 1–2 cm proximal to apex of FT; ACB: 1–2 cm distal to the apex of FT.
Technique	Injected adjacent to the FA targeting SN within FT	Targeting SN in the mid-thigh without specific fascial precision	FTB component: Indirect, not within distal FT; between STM and VMM, targeting NVM and SN lateral to FA. ACB: Especially in proximal AC to avoid proximity to the sterile surgical field. Administered only after the FT injection
Target nerves	SN ± NVM	SN	FTB: SN + NVM; ACB: SN and perivascular under the VAM
Volume of LA	15-20 ml	15-20 ml	10-20 ml + 10-20 ml (split between sites)
Approach precision	Anatomical approximation	Anatomical approximation	Real-time ultrasound-guided precision from the apex of FT with clear fascial planes
Coverage	Partial anterior/medial knee	Intraarticular and partial anterior knee	Comprehensive (anterior, posterior, intraarticular, and extraarticular)
Procedure-specific	No	No	Yes - tailored to TKA pain pattern
Opioid-sparing	Moderate	Low	High
Motor-sparing	Yes	Yes	Yes (preserved in both components)

Patient assessment and data collection

The primary outcome, the quadriceps strength (QS) in the operated limb, was assessed every six hours postoperatively until discharge using a 6-point neurological scale (0-5), where 0 indicated no contraction, and 5 indicated normal strength [26]. Buckling has been defined as a sudden, unintentional loss of postural stability and balance, as observed by staff, when a patient requires support to prevent a fall [27].

**Figure 2:**
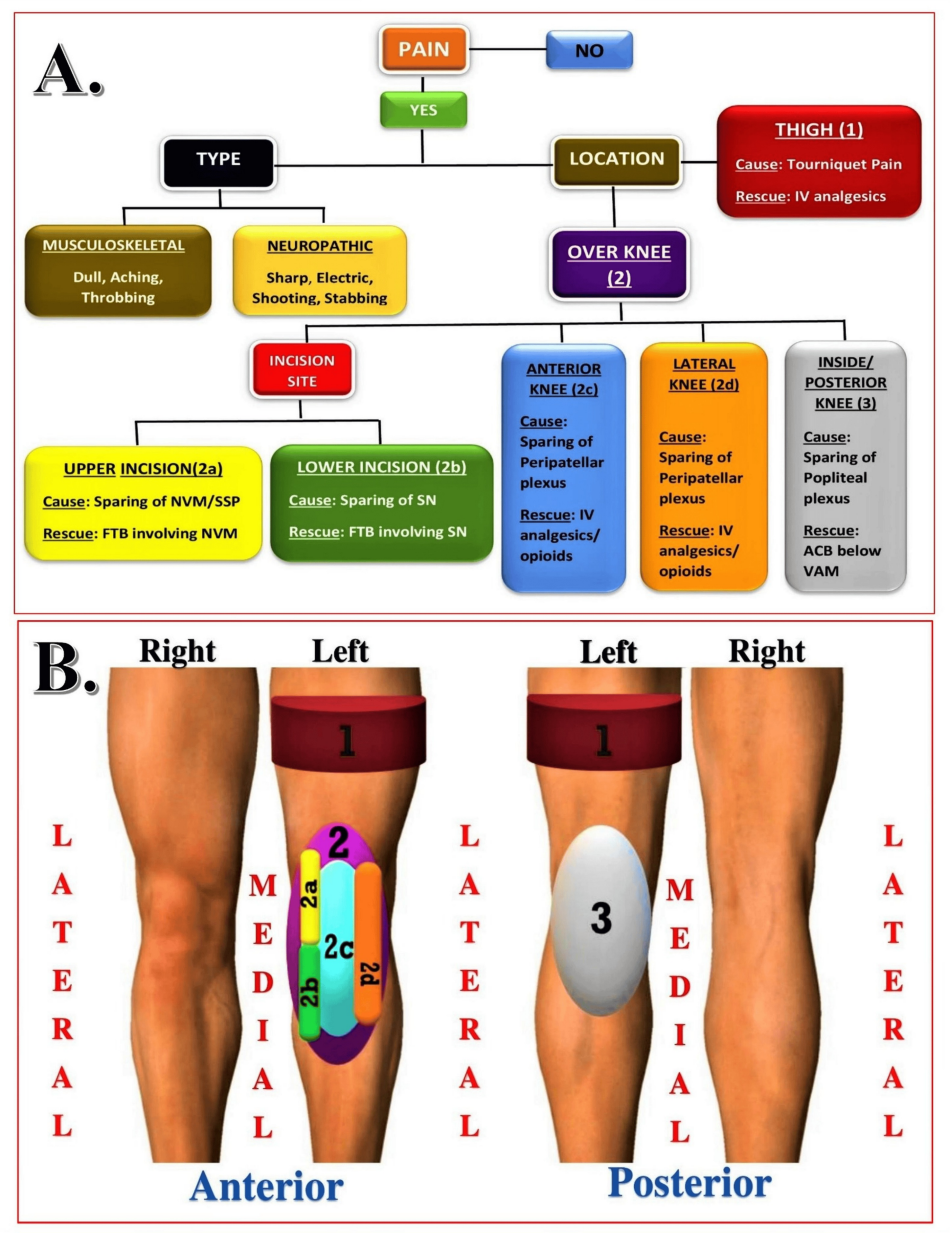
Postoperative Pain Assessment and Rescue Analgesia Protocol. Rescue Analgesia According to Pain Location: Postoperative rescue analgesia was tailored based on specific anatomical sites of knee pain. Pain Location Mapping: Pain was coded systematically to guide targeted analgesia: Code 1 represented pain localized to the thigh; Code 2 referred to pain in the anterior knee region, further subclassified into 2A (upper part of the incision), 2B (lower part of the incision), 2C (central anterior knee), and 2D (lateral knee); Code 3 indicated pain in the posterior knee region; and the designation “mixed locations” was used when multiple areas were simultaneously painful. Rescue analgesia was initiated for VAS > 4 using opioids, in line with the study protocol that prioritized pharmacologic over procedural interventions to optimize comfort based on pain mapping. IV, intravenous; NVM, nerve to vastus medialis; SSP, subsartorial plexus; FTB, femoral triangle block; SN, saphenous nerve; ACB, adductor canal block; VAM, vastoadductor membrane. Source: Adapted from Sonawane et al. under Creative Commons Attribution License [21].

One of the secondary outcomes was the analgesic efficacy of each block, evaluated by comparing static and dynamic postoperative pain scores (SPS and DPS) between groups using the visual analog scale (VAS) at regular intervals until discharge. Other secondary outcomes included the estimation of additional postoperative opioid (fentanyl) consumption between groups till discharge, recorded and converted to intravenous morphine equivalents (ME in mg). Rescue analgesia was administered based on postoperative pain mapping (Figures 2A, 2B) when VAS >4 using 1-3 codes (1 = thigh, 2 = anterior knee (2A = upper part of incision, 2B = lower part of incision, 2C = anterior knee, 2D = lateral knee), 3 = posterior knee, and mixed locations). However, to align with the study design, rescue opioids were preferred over rescue blocks as per the pain mapping protocol to ensure patient comfort and effective pain relief. The sensory block duration was defined as the time from block completion to the first request for analgesia or VAS ≥ 4. Patient satisfaction was assessed at discharge using a 3-point scale (1 = unsatisfied, 2 = satisfied, 3 = fully satisfied), and any postoperative side effects or complications were documented.

Statistical analysis

Data were recorded using Microsoft Excel and analyzed with IBM SPSS Statistics for Windows, Version 24 (Released 2016; IBM Corp., Armonk, New York, United States). Continuous variables were summarized as means ± standard deviations (SD) or medians with interquartile ranges (IQR), depending on the normality of the data. Categorical variables were expressed as frequencies and percentages. Normality was assessed using the Shapiro-Wilk test. For intergroup comparisons, one-way ANOVA was used for normally distributed data, with Tukey’s post-hoc test for pairwise analysis. Categorical variables were analyzed using the chi-square test or Fisher’s exact test, depending on expected frequencies.

Non-parametric comparisons were performed using the Kruskal-Wallis test, followed by Mann-Whitney U tests with Bonferroni correction where applicable. Multiplicity arising from multiple pairwise comparisons (DSB vs. FTB and DSB vs. ACB) was controlled using a Bonferroni adjustment. A p-value < 0.05 was considered statistically significant for all comparisons. Graphical data, including line diagrams and violin plots, were used to visualize distribution patterns and enhance the interpretation of key outcomes.

## Results

Patient demographics

**Figure 3:**
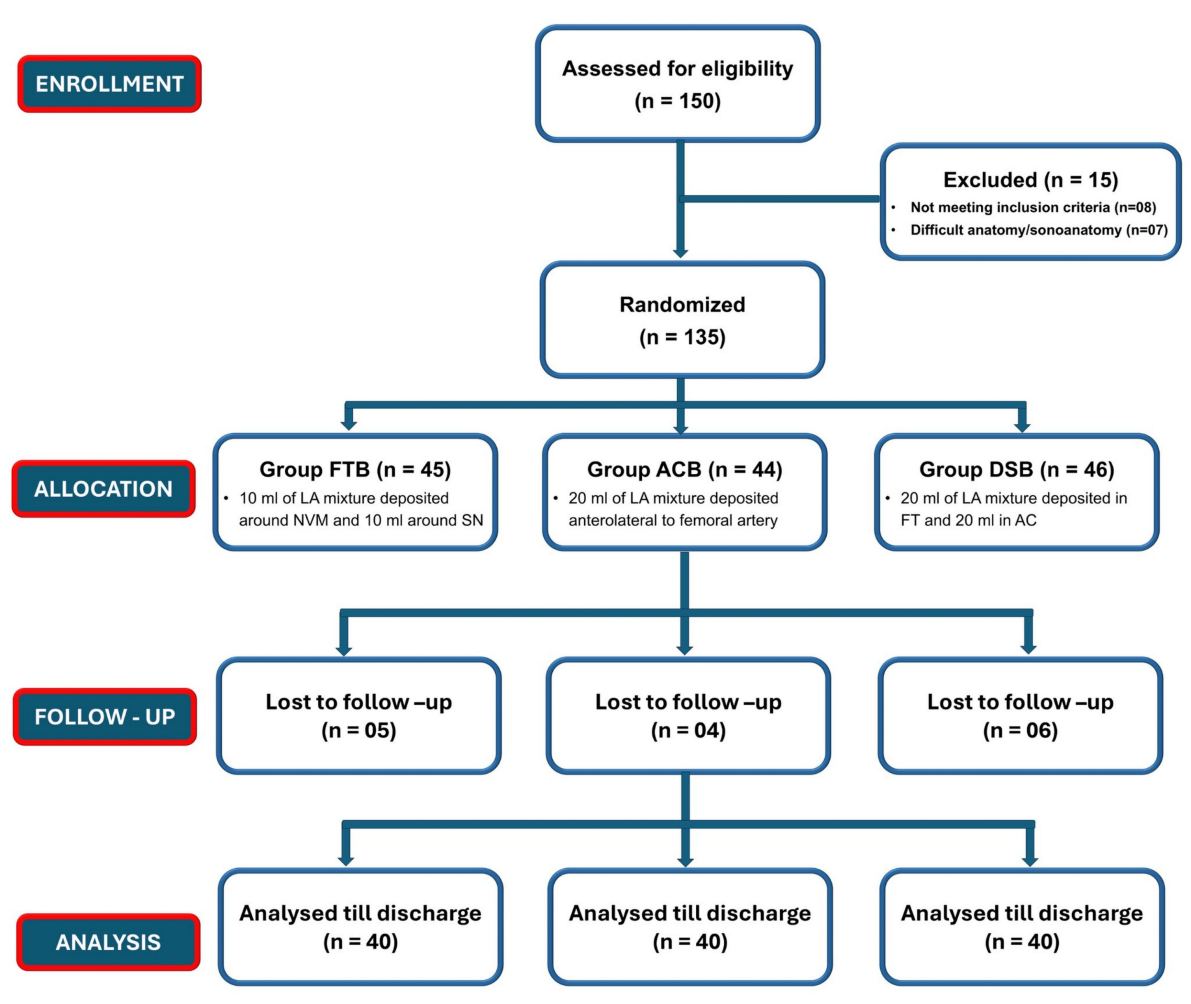
CONSORT Flow Diagram Illustrating Patient Enrollment, Randomization, Group Allocation, Follow-up, and Final Analysis. DSB, dual subsartorial block; FT, femoral triangle; AC, adductor canal; LA, local anesthetic; FTB, femoral triangle block; ACB, adductor canal block.

The assignment of the patients to study groups is shown in Figure 3. A total of 120 patients were randomized equally into three groups (FTB, ACB, DSB). Baseline characteristics, including age (p = 0.770), gender (χ² = 0.077, p = 0.962), and BMI (p = 0.944), were statistically comparable across groups, confirming demographic balance (Table 5).

**Table 5:** Characteristics of the Study Population. Data are presented as n (%) and Mean ± SD, as appropriate. p-values <0.05 were considered statistically significant. Demographics: Age, sex, and BMI were comparable across groups (p > 0.05). FTB, femoral triangle block; ACB, adductor Canal Block; DSB, dual subsartorial block; BMI, body mass index; SD, standard deviation; IQR, interquartile range; p, P-value (statistical significance).

Variable	FTB (n=40)	ACB (n=40)	DSB (n=40)	Test statistic	p-value
Sex (Male)	13 (34.2%)	13 (34.2%)	12 (31.6%)	χ² = 0.077	0.962
Sex (Female)	27 (32.9%)	27 (32.9%)	28 (34.1%)		
Age (years)	66.55 ± 5.86	67.33 ± 5.60	66.48 ± 5.98	F = 0.262	0.77
BMI (kg/m²)	31.10 ± 2.68	31.20 ± 2.90	30.97 ± 3.49	F = 0.058	0.944

Post-block quadriceps strength

**Figure 4:**
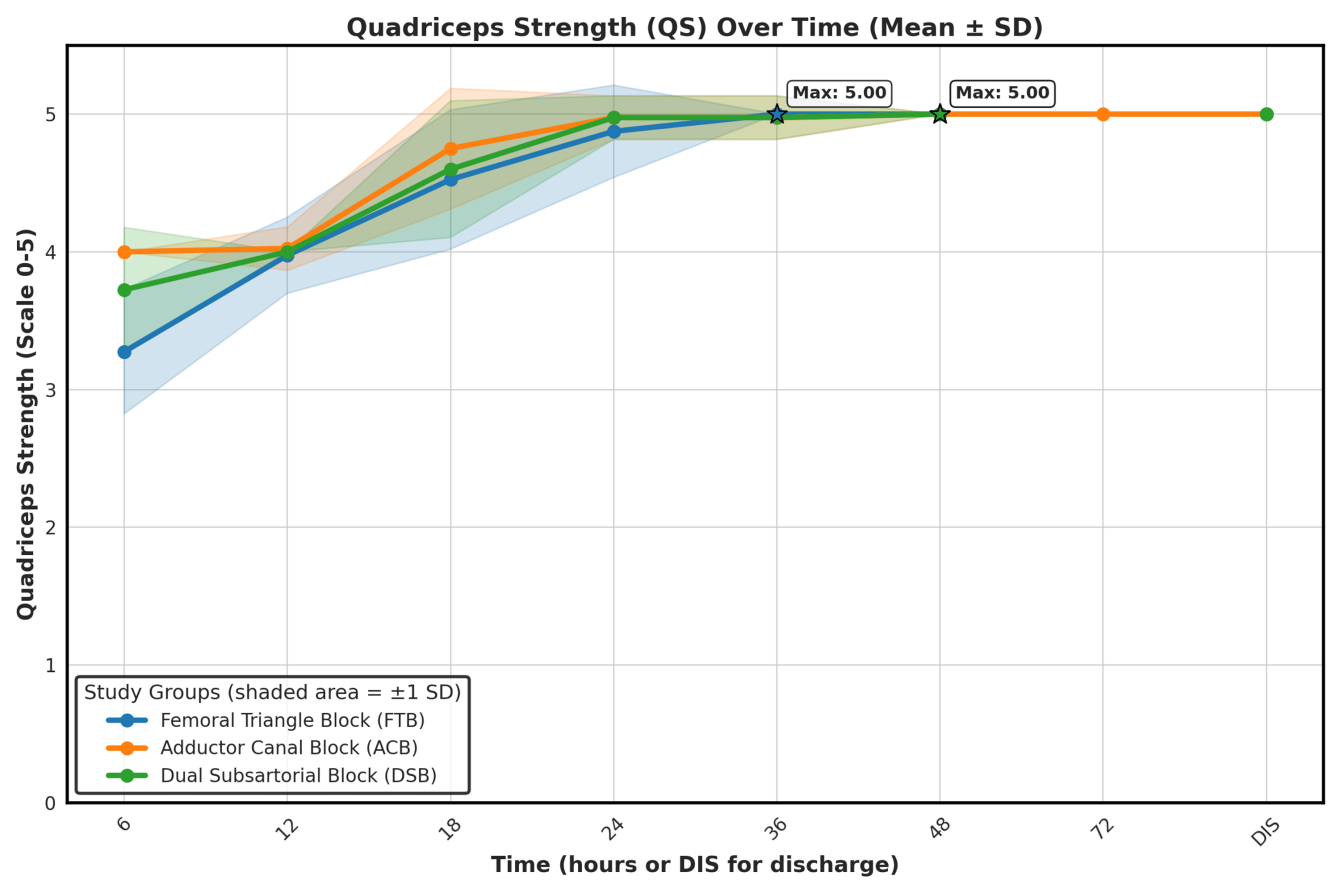
Quadriceps Strength (QS) Recovery over Time Following Three Regional Anesthesia Techniques. Data are presented as mean ± SD. Statistical significance was considered at p < 0.05. Mean quadriceps strength (on a 0–5 scale) is plotted at predefined postoperative intervals (6, 12, 18, 24, 36, 48, 72 hours, and discharge). The shaded area around each line represents ±1 standard deviation (SD). All groups demonstrated progressive improvement in muscle strength over time, with the DSB and ACB groups achieving faster early recovery. By 36 hours, most patients across all groups had regained maximal quadriceps strength (score = 5.00), maintained through discharge. QS, quadriceps strength; SD, standard deviation; FTB, femoral triangle block; ACB, adductor canal block; DSB, dual subsartorial block; DIS, discharge.

The primary outcome of the study was to assess QS following block administration (Table 6, Figure 4). At six hours postoperatively, ACB (4.00 ± 0.00) and DSB (3.73 ± 0.45) groups showed significantly higher quadriceps strength compared to FTB (3.27 ± 0.45) (p < 0.001). From 12 hours onward, no significant differences were noted (p > 0.05), indicating equivalent motor preservation among all three blocks.

**Table 6:** Post-block Quadriceps Strength Over Time. Early motor preservation was better with DSB and ACB; full recovery was achieved in all groups by the time of discharge. Values represented as mean ± SD. p-values <0.05 were considered statistically significant. FTB, femoral triangle block; ACB, adductor canal block; DSB, dual subsartorial block; QS, quadriceps strength; SD, standard deviation; P-value (statistical significance).

Variable	FTB (n=40)	ACB (n=40)	DSB (n=40)	Test statistic/p-value	
QS 6	3.27 ± 0.45	4.00 ± 0.00	3.73 ± 0.45	0	
QS 12	3.98 ± 0.28	4.03 ± 0.16	4.00 ± 0.00	0.479	
QS 18	4.53 ± 0.51	4.75 ± 0.44	4.60 ± 0.50	0.108	
QS 24	4.88 ± 0.33	4.97 ± 0.16	4.97 ± 0.16	0.089	
QS 36	5.00 ± 0.00	4.97 ± 0.16	4.97 ± 0.16	0.608	
QS 48	5.00 ± 0.00	5.00 ± 0.00	5.00 ± 0.00	1	
QS DIS	5.00 ± 0.00	5.00 ± 0.00	5.00 ± 0.00	1	

Analgesic efficacy

**Figure 5:**
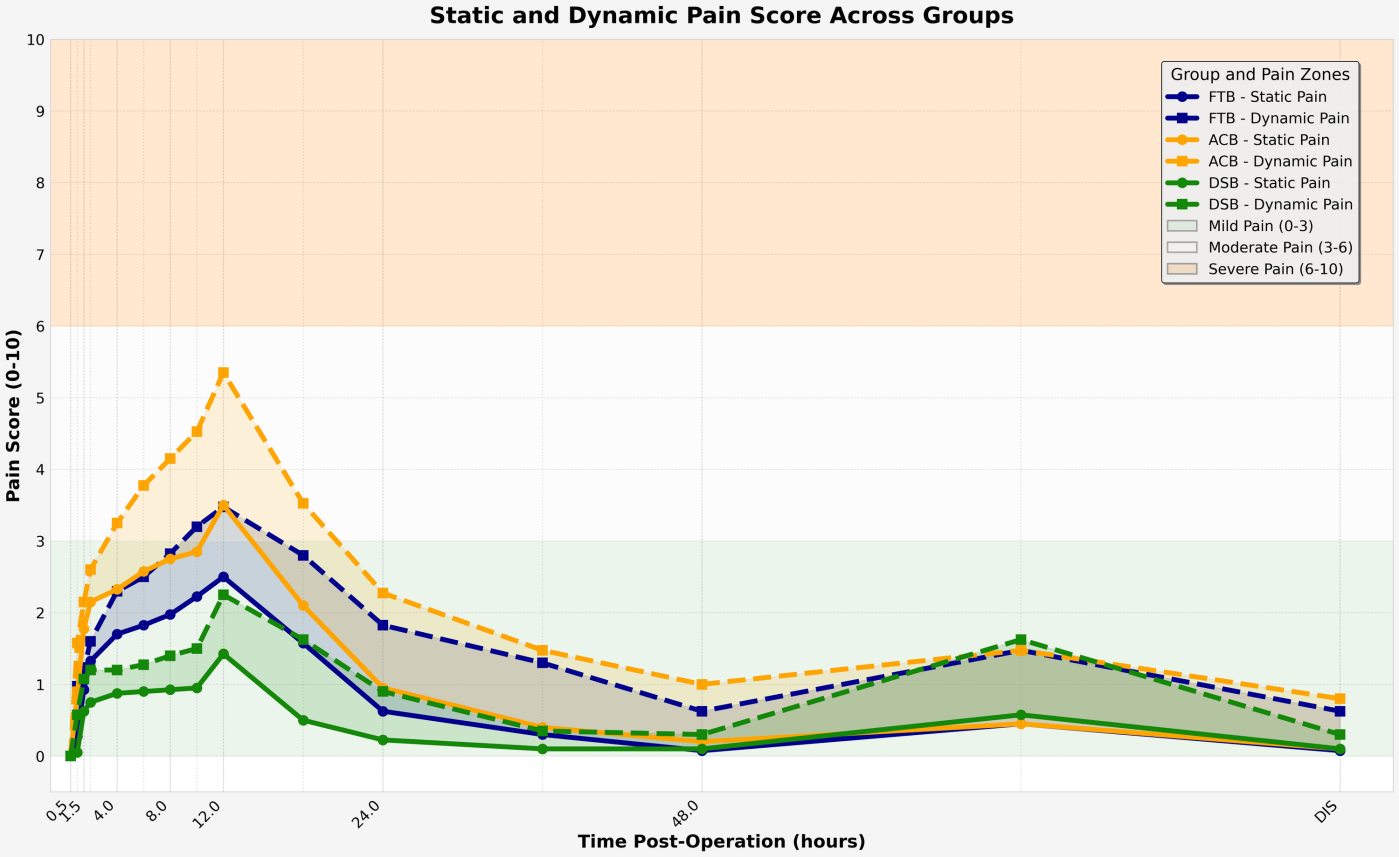
Static and Dynamic Pain Scores over Time Measured Using the Visual Analog Scale (VAS) across Study Groups. Data are presented as mean values, and statistical significance is considered at p < 0.05. This line graph illustrates the temporal progression of static (SPS) and dynamic (DPS) pain scores from 0.5 to 72 hours and at discharge (DIS) in patients who underwent total knee arthroplasty (TKA) under three different block techniques. Pain scores (VAS, 0–10) were recorded at multiple postoperative time points under static and dynamic conditions. Background shading represents pain severity: green (mild, VAS 0–3), white (moderate, VAS 3–6), and orange (severe, VAS 6–10). The DSB group consistently maintained pain scores within the mild range across all timepoints, especially in the first 24 hours. The ACB group showed the highest early postoperative pain, especially during movement, with scores reaching moderate to severe levels. The FTB group demonstrated intermediate efficacy, with scores largely within the mild to moderate range. VAS, visual analog scale; FTB, femoral triangle block; ACB, adductor canal block; DSB, dual subsartorial block; DIS, discharge.

The secondary objective of the study was to evaluate the analgesic efficacy of each block (Table 7, Figure 5). DSB consistently demonstrated lower static and dynamic pain scores, especially within the first 24 hours (p < 0.001). While static pain scores equalized by 48 hours (p > 0.05), dynamic scores remained lower in DSB (p < 0.001). ACB showed the highest pain levels, with peak pain around 12 hours.

**Table 7:** Differences in Static Pain Score (SPS) and Dynamic Pain Score (DPS) across Blocks. DSB had significantly lower static (SPS) and dynamic (DPS) pain, particularly in the early postoperative period (p < 0.05). Values represented as median (IQR). p-values <0.05 were considered statistically significant. FTB, femoral triangle block; ACB, adductor canal block; DSB, dual subsartorial block; SPS, static pain score; DPS, dynamic pain score; VAS, visual analog scale; IQR, interquartile range; DIS, discharge; p, P-value (statistical significance).

Variable	FTB (n=40)	ACB (n=40)	DSB (n=40)	Test statistic/p-value	
SPS 1	0 (0.11 - 0.44)	1 (0.58 - 1.12)	0 (-0.02 - 0.12)	0	
SPS 1.5	1 (0.66 - 1.19)	2 (1.42 - 2.13)	1 (0.44 - 0.81)	0	
SPS 2	1 (1.02 - 1.63)	2 (1.81 - 2.49)	1 (0.55 - 0.95)	0	
SPS 4	2 (1.4 - 2)	2 (1.96 - 2.69)	1 (0.66 - 1.09)	0	
SPS 6	2 (1.5 - 2.15)	3 (2.2 - 2.95)	1 (0.67 - 1.13)	0	
SPS 8	2 (1.63 - 2.32)	3 (2.41 - 3.09)	1 (0.7 - 1.15)	0	
SPS 10	2 (1.86 - 2.59)	3 (2.49 - 3.21)	1 (0.72 - 1.18)	0	
SPS 12	3 (2.1 - 2.9)	4 (3.16 - 3.84)	2 (1.07 - 1.78)	0	
SPS 18	1 (1.29 - 1.86)	2 (1.81 - 2.39)	0 (0.3 - 0.7)	0	
SPS 24	0.5 (0.4 - 0.85)	1 (0.76 - 1.14)	0 (0.09 - 0.36)	0	
SPS 36	0 (0.15 - 0.45)	0 (0.24 - 0.56)	0 (0 - 0.2)	0.01	
SPS 48	0 (-0.01 - 0.16)	0 (0.07 - 0.33)	0 (0 - 0.2)	0.2	
SPS DIS	0 (-0.01 - 0.16)	0 (0 - 0.2)	0 (0 - 0.2)	0.91	
DPS 1	1 (0.74 – 1.21)	2 (1.30 – 1.85)	1 (0.40 – 0.75)	0	
DPS 1.5	1 (0.93 – 1.47)	2 (1.79 – 2.51)	1 (0.89 – 1.26)	0	
DPS 2	1 (1.30 – 1.90)	2 (2.28 – 2.92)	1 (0.99 – 1.41)	0	
DPS 4	2 (2.00 – 2.60)	3 (2.92 – 3.58)	1 (0.99 – 1.41)	0	
DPS 6	2.5 (2.19 – 2.81)	4 (3.46 – 4.09)	1 (1.06 – 1.49)	0	
DPS 8	3 (2.44 – 3.21)	4 (3.77 – 4.53)	1.5 (1.16 – 1.64)	0	
DPS 10	3 (2.80 – 3.60)	5 (4.14 – 4.91)	2 (1.26 – 1.74)	0	
DPS 12	3 (3.01 – 3.94)	5 (5.03 – 5.67)	2 (1.88 – 2.62)	0	
DPS 18	3 (2.51 – 3.09)	4 (3.12 – 3.93)	2 (1.35 – 1.90)	0	
DPS 24	2 (1.55 – 2.10)	2 (1.98 – 2.57)	1 (0.67 – 1.13)	0	
DPS 36	1 (1.08 – 1.52)	2 (1.25 – 1.70)	0 (0.18 – 0.52)	0	
DPS 48	1 (0.41 – 0.84)	1 (0.78 – 1.22)	0 (0.15 – 0.45)	0	
DPS DIS	1 (0.41 – 0.84)	1 (0.62 – 0.98)	0 (0.15 – 0.45)	0.001	

Pain mapping

**Figure 6:**
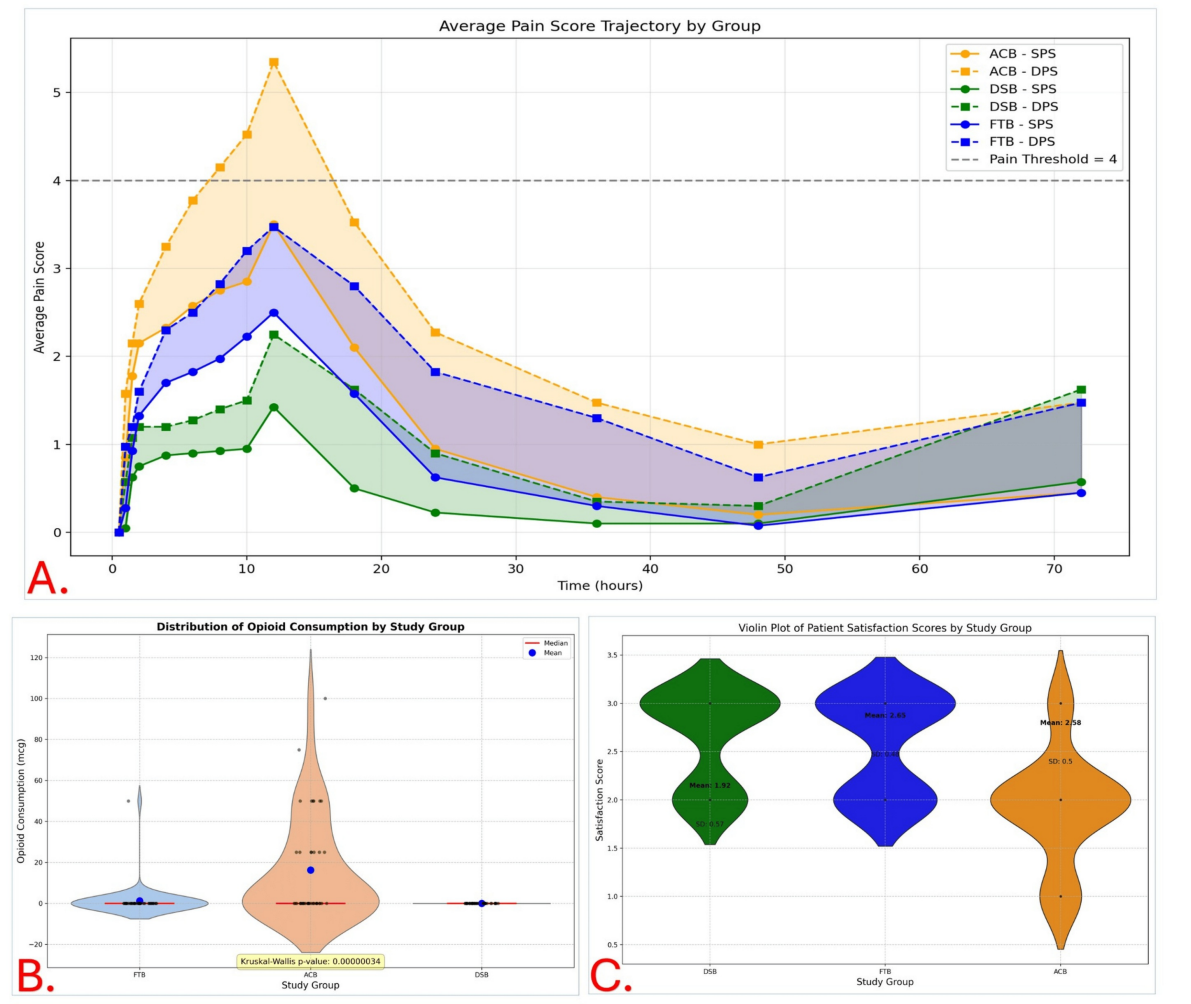
Pain Trajectory, Opioid Use, and Patient Satisfaction by Study Group. All data are presented as mean ± SD with shaded area or violin distribution plots. Statistical significance is defined at p < 0.05. A. Pain Score Trajectories: VAS scores (0–10) over time under static (SPS) and dynamic (DPS) conditions, with shaded areas between each group's SPS and DPS lines representing the difference between pain at rest and movement. Dashed black line indicates pain threshold (VAS = 4). The DSB group consistently reported the lowest pain scores, especially during the early postoperative period. The ACB group exhibited the highest pain levels, often exceeding the pain threshold of 4, particularly in dynamic assessments. B. Opioid Consumption: Violin plot comparing postoperative opioid consumption (mcg) across groups. The DSB group required no opioids, while the ACB group had significantly higher usage. Kruskal-Wallis test confirmed significant differences between groups (p < 0.000001). C. Patient Satisfaction: Violin plot showing distribution of satisfaction scores (scale 1–3) across groups. DSB (mean 2.62) and FTB (mean 2.65) groups reported higher satisfaction, whereas the ACB group had the lowest (mean 1.93 ± 0.57). FTB, femoral triangle block; ACB, adductor canal block; DSB, dual subsartorial block; SPS, static pain score; DPS, dynamic pain score; VAS, visual analog scale.

Pain mapping (Figure 2) was routinely performed before administering rescue opioids whenever pain scores exceeded 4 (Figure 6A), as part of our standard-of-care protocol to identify potential regions of inadequate analgesic coverage (“block sparing”). Pain mapping (Table 8) revealed the highest incidence in ACB (n = 15), mainly at the upper incision (73.3%). FTB had one report (lateral knee), and DSB had none. Statistical comparisons confirmed significant differences (χ² = 30.43, p < 0.001; Fisher’s exact test: ACB vs DSB, p = 0.00001).

**Table 8:** Pain Mapping (Codes and Locations). The ACB group showed more localized and mixed-site pain, especially at upper incision sites (2A) (χ² = 30.43, p < 0.001). FTB, femoral triangle block; ACB, adductor canal block; DSB, dual subsartorial block; χ², Chi-square test; p, P-value (statistical significance).

Variable	FTB (n=40)	ACB (n=40)	DSB (n=40)	Test statistic/p-value	
1 (Thigh)	0	0	0	χ² = 30.43, p < 0.001	
2A (Upper incision)	0	11	0		
2B (Lower incision)	0	0	0		
2C (Anterior knee)	1	0	0		
2D (Lateral knee)	0	2	0		
3 (Posterior knee)	0	0	0		
Mixed locations	0	2	0		

Post-block additional (rescue) opioid consumption

The secondary outcome was to assess rescue opioid requirements among the three block groups (Table 9). Opioid requirements varied significantly: ACB (37.5% patients, mean 16.25 mg), FTB (2.5%, 1.25 mg), and DSB (0%, 0 mg). ANOVA (p = 0.00000292) and Kruskal-Wallis (p = 0.00000034) confirmed group differences. Post-hoc tests showed significantly higher use in ACB vs both DSB and FTB. Violin plots (Figure 6B) illustrated the skewed distribution of opioid use, with most patients having zero requirements but a cluster of moderate-to-high usage in the ACB group.

**Table 9:** Summary of Rescue Opioid Consumption Across Groups. No opioid requirement in DSB group; highest usage observed in ACB (p < 0.001 vs DSB/FTB). Mean opioid use reported in morphine equivalents (ME). FTB, femoral triangle block; ACB, adductor canal block; DSB, dual subsartorial block; ME, morphine equivalent; p, P-value (statistical significance).

Variable	FTB (n=40)	ACB (n=40)	DSB (n=40)	Test statistic/p-value	
Patients requiring opioids (n, %)	1 (2.5%)	15 (37.5%)	0/40 (0%)	p < 0.001 (ACB vs FTB & DSB)	
Total Fentanyl (mcg)	50	650	0		
Total Morphine (mg)	5	65	0	Not Significant (FTB vs DSB)	
Mean opioid use (mg ME)	1.25	16.25	0		

Patient satisfaction score

DSB showed the highest satisfaction (2.65 ± 0.48), followed by FTB (2.58 ± 0.50), and ACB (1.93 ± 0.57). Group differences were significant (ANOVA and Kruskal-Wallis, p < 0.001). ACB had broader variability and more dissatisfaction (Table 10). Violin plots (Figure 6C) illustrated a broader, lower-end distribution in ACB, while DSB and FTB showed tighter clustering around higher satisfaction scores, indicating greater consistency and patient approval.

**Table 10:** Association Between Patient Satisfaction and Block. All groups reported high satisfaction; DSB had the highest mean score (2.65 ± 0.48). Data are presented as n (%) and Mean ± SD, as appropriate. P-values <0.05 were considered statistically significant. FTB, femoral triangle block; ACB, adductor canal block; DSB, dual subsartorial block; SD, standard deviation; p, P-value (statistical significance).

Variable	FTB (n=40)	ACB (n=40)	DSB (n=40)	Test statistic/p-value	
Unsatisfied	5 (12.5%)	2 (5.0%)	1 (2.5%)	0.415	
Satisfied	19 (47.5%)	18 (45.0%)	21 (52.5%)		
Very Satisfied	16 (40.0%)	20 (50.0%)	18 (45.0%)		
Mean ± SD	2.58 ± 0.50	1.93 ± 0.57	2.65 ± 0.48		

Block duration and effectiveness

Block duration, defined as the time from block administration to the first request for rescue opioids due to VAS >4. It was the shortest in the ACB group, where breakthrough pain and a wide SPS-DPS gap emerged within 4-6 hours postoperatively (Table 11). ACB patients had a mean block duration of 16.3 ± 8.76 hours (median 12 hours), reflecting high variability and inconsistent analgesia. The FTB group demonstrated moderate efficacy, characterized by delayed pain onset, smaller SPS-DPS differences, and the need for rescue opioids in only one patient (block duration: 8 hours). Statistical analysis confirmed overall group differences (ANOVA, p = 0.0012), with significant pairwise differences between ACB and FTB (p = 0.0174) and ACB and DSB (p = 0.0042), but not between FTB and DSB (p = 0.3235).

**Table 11:** Analgesia Summary with Block Duration and Rescue Opioids. DSB provided >24 hours of analgesia - significantly longer than ACB. Data are presented as Mean ± SD, as appropriate. P-values <0.05 were considered statistically significant. FTB, femoral triangle block; ACB, adductor canal block; DSB, dual subsartorial block; SD, standard deviation; ME, morphine equivalent; p, P-value (statistical significance).

Variable	FTB (n=1/40)	ACB (n=15/40)	DSB (n=0/40)	Test statistic/p-value	
Block duration (Mean ± SD, hrs)	8 ± NA	16.3 ± 8.76	>24 ± NA	FTB vs ACB p < 0.0174	
Median block duration (hrs)	8	12	>24	FTB vs DSB p < 0.3235	
Mean opioid consumption (ME)	1.25	16.25	0	ACB vs DSB p < 0.0042	

## Discussion

Pursuing optimal RA techniques for TKA remains a critical research focus due to the need for effective analgesia, opioid reduction, and motor preservation. In this comparative study, we evaluated three RA techniques (DSB, FTB, and ACB) and found that DSB consistently outperformed the others in achieving this balance. Patients in the DSB group exhibited no motor weakness throughout the postoperative period, reaffirming its motor-sparing profile alongside FTB and ACB. Additionally, DSB consistently maintained pain scores below the clinical threshold of VAS ≥ 4, with no requirement for rescue opioids and block durations exceeding 24 hours. In comparison, ACB patients reported the earliest breakthrough pain (within 4-6 hours) and the highest variability in pain scores, accompanied by a higher need for rescue opioids (mean block duration: 16.3 ± 8.76 hours). FTB showed intermediate performance. ANOVA confirmed significant overall differences (p = 0.0012), with notable pairwise significance between ACB and both DSB (p = 0.0042) and FTB (p = 0.0174). These results reflect DSB’s clinical advantage in analgesic reliability, which naturally extends into patient experience. Patient satisfaction mirrored clinical outcomes: highest in DSB, followed by FTB, with ACB lowest (p < 0.001). These results reflect the correlation between better pain control and higher patient-reported satisfaction, supporting DSB as a technique tailored for procedure-specific analgesia in TKA.

Procedure-specific analgesia

**Figure 7:**
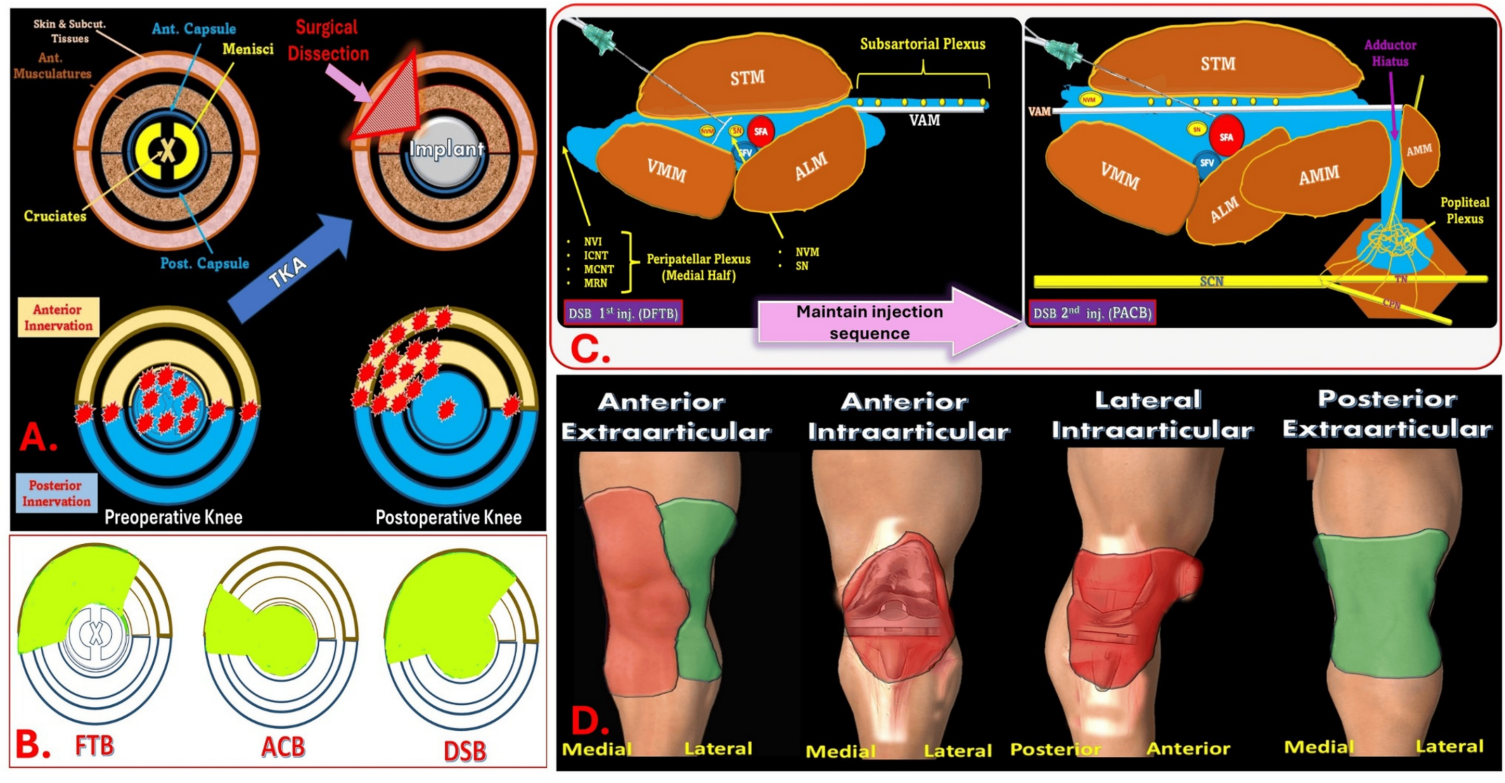
Procedure-Specific Characteristics of the Dual Subsartorial Block (DSB). A. Cross-sectional view of knee innervation. Yellow and blue represent anterior and posterior innervations, respectively. Red stars mark common pain-generating zones following surgical dissection and implant placement. B. Circular diagram comparing the sensory coverage of FTB, ACB, and DSB. Green-shaded regions denote effective blockade, with DSB showing the most comprehensive coverage. C. Dual-injection technique of DSB: distal femoral triangle and proximal adductor canal, covering subsartorial, peripatellar, and popliteal plexuses. D. Illustrates analgesic distribution across key zones - anterior extra-and intra-articular, lateral intra-articular, and posterior extra-articular. The first injection of DSB targets the SN and NVM, with LA spreading proximally within the femoral triangle and distally into the adductor canal beneath the sartorius and above the VAM. This provides coverage of the SN, NVM, medial peripatellar plexus, and subsartorial plexus. The second injection fills the remaining AC via a perivascular approach beneath the VAM. Residual LA may spread into the popliteal fossa through the adductor hiatus, engaging the popliteal plexus. The overall spread covers the anteromedial knee, tibial tuberosity, medial retinaculum, and intra-articular components, excluding the anterolateral skin (lateral peripatellar plexus) and posterior knee (posterior femoral cutaneous nerve). Yellow-colored areas, anterior knee innervation; blue-colored areas, posterior knee innervation; red star-like dots, pain-generating zones; green-colored areas, analgesic coverage; FTB, femoral triangle block; ACB, adductor canal block; DSB, dual subsartorial block; DFTB, distal femoral triangle block; PACB, proximal adductor canal block; TKA, total knee arthroplasty; SN, saphenous nerve; NVM, nerve to vastus medialis; VAM, vastoadductor membrane; LA, local anesthetic. Source: A and B were adapted from Sonawane et al. [23].  C and D were created by the first author KS.

TKA presents a unique postoperative pain profile where intra-articular pain generators diminish over time while extra-articular sources, such as the incision site, medial retinaculum, periosteal rim, joint capsule remnants, and microfractures, dominate (Figure 7A) [22,24,28]. A procedure-specific RA technique must address anterior, posterior, and intra-articular knee innervation. ACB primarily targets the SN directly and the popliteal plexus indirectly, offering partial anterior and posterior coverage [29]. FTB provides anterior knee analgesia but lacks posterior spread, resulting in incomplete coverage [29,30]. DSB combines the benefits of ACB and FTB, ensuring comprehensive analgesic coverage of anterior, posterior, and intra-articular pain generators of the knee joint (Figure 7B). This was reflected in the DSB group’s significantly lower SPS and DPS across all time points within the first 24 hours (p < 0.001). This makes DSB a more suitable option for addressing the diverse pain generators in TKA.

Analgesic efficacy and technical accuracy

Beyond anatomical coverage, the hallmark of DSB is its consistent analgesic efficacy. Our findings align with existing literature supporting DSB’s superior and reliable pain control, with pain scores consistently below 3 in the early postoperative period, critical for early mobility and rehabilitation [21]. In contrast, studies comparing FTB and ACB have shown mixed results, often due to mislabeling and technical inconsistencies [31]. Some reported better analgesia with FTB [32], while others favored ‘true’ ACB [33]. Chuan et al. found no significant differences in pain relief or opioid consumption between the two techniques [34], highlighting inconsistencies in functional outcomes across studies.

These inconsistencies may arise from variations in anatomical labeling and procedural execution. One major contributing factor to this outcome discrepancy is the incorrect administration of ACB within the FT region, leading to misclassification and misinterpretation of results [31,35,36]. However, some studies labeled mid-thigh injections as ACB when these were actually FTBs anatomically, leading to inconsistencies and confusion in reported outcomes [35,36]. Despite clear anatomical landmarks, errors in block placement have led to a cycle of misinformation, with many RCTs and meta-analyses failing to distinguish true ACB from misapplied FTB. This misrepresentation skews comparative outcomes and compromises clinical decision-making, block selection, and pain management strategies.

The persistence of inaccurate procedural descriptions in the literature continues to create a “Triangle and Tunnel of Confusion” (FT and AC), making it imperative to establish standardized nomenclature and precise technique descriptions. To address this, precise sonographic identification of the apex of the FT, seen as the characteristic "sign of 3", is essential for the correct placement of injections in both the FT and AC territories. DSB, by design, requires precise sonographic guidance, minimizing errors and enhancing both accuracy and reproducibility. Our study emphasizes the importance of standardized terminology and techniques to ensure meaningful comparisons across research and clinical practice. Clarifying these standards is vital for improving clinical decision-making and advancing RA practice.

Anatomical coverage and pain mapping insights

A major limitation of ACB and FTB lies in their incomplete analgesic reach - either missing intra-articular or extraarticular pain sources. Our pain mapping revealed that 73% of ACB patients experienced pain at the upper incision site, a finding absent in the DSB group. To overcome such gaps, combination techniques like ACB + Infiltration between the Popliteal Artery and the Capsule of the Knee (IPACK) [37,38], FTB + IPACK [39], and ACB/FTB with genicular or popliteal plexus blocks [40-42] have been explored for broader coverage and opioid-sparing effects. However, many still fall short in addressing extraarticular pain comprehensively. In contrast, DSB offers comprehensive, full-spectrum analgesia with superior opioid-sparing efficacy. These findings align with the recommendations of Marty et al. [43], who advocate for combining proximal and distal nerve blocks to optimize MMA.

Opioid-sparing impact

Opioids remain a traditional mainstay in perioperative TKA pain management, often utilized to address chronic pain components from OA [44]. In our protocol, a low-dose transdermal buprenorphine patch (5-10 mg) was included for background pain relief [45], while rescue opioids were administered only when necessary, ensuring patients remained pain-free without routine supplemental nerve blocks. Reducing opioid consumption is not only a pharmacologic goal but also a patient safety priority, given its association with prolonged hospital stays, side effects, and delayed rehabilitation. DSB exhibited a striking opioid-sparing effect, with no rescue opioid use in any patient. In contrast, the ACB group had the highest morphine-equivalent consumption (mean: 16.25 mg), while FTB patients required minimal supplementation (1.25 mg). This disparity underscores both the depth and duration of analgesia offered by DSB.

By integrating dual injections (Figure 7C), DSB effectively targets all relevant nociceptive pathways (Figure 7D), significantly reducing opioid reliance while preserving motor function. This balanced approach enhances pain relief, supports early mobilization, and aligns well with ERAS goals, making DSB a reliable, opioid-sparing approach for improved patient outcomes.

Motor-sparing precision of DSB

**Figure 8:**
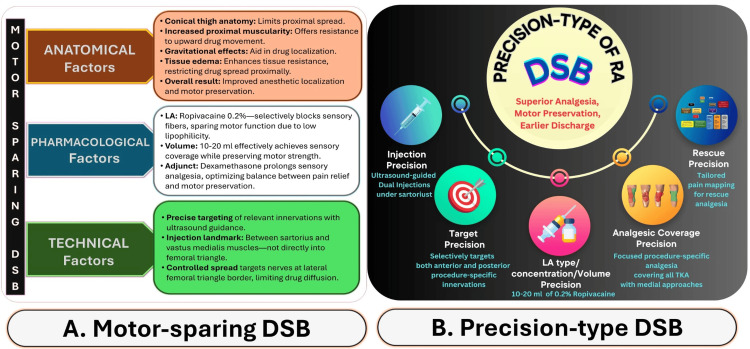
Motor-Sparing and Precision Characteristics of the DSB. A. Factors Supporting Motor-Sparing: Anatomy: Conical thigh shape, proximal muscle bulk, and tissue edema restrict proximal spread. Pharmacology: Use of 0.2% ropivacaine selectively blocks sensory fibers while sparing motor function. Technique: Ultrasound-guided injection between the sartorius and vastus medialis ensures precise targeting of sensory nerves with minimal motor involvement B. Precision-Type DSB Framework: A procedure-specific approach built on five key precision elements: Injection Precision: Dual ultrasound-guided injections beneath the sartorius. Target Precision: Selective delivery to anterior and posterior sensory pathways relevant to TKA. LA Precision: Controlled volumes (10–20 ml of 0.2% ropivacaine) for effective sensory blockade. Coverage Precision: Focused analgesia optimized for TKA with medial surgical approaches. Rescue Precision: Enables individualized pain management using pain-location mapping. This integrated strategy enhances analgesia, minimizes motor block, and facilitates early mobilization and discharge post-TKA. DSB, dual subsartorial block; TKA, total knee arthroplasty; LA, local anesthetic. Source: This image was created by the first author KS.

Effective pain control and early ambulation are critical for optimal TKA outcomes [46], as quadriceps weakness can lead to falls, delayed rehabilitation, and increased healthcare costs. Our study observed no falls or significant motor impairment, validating the motor-sparing profile of all three techniques. However, DSB offered the best balance between superior pain control and mobility. This advantage stems from DSB’s unique integration of anatomical precision, technical refinements, and pharmacological considerations (Figure 8A). Such precision-driven enhancements (Figure 8B) improve analgesia and support early discharge, hallmarks of effective ERAS implementation.

Strengths, limitations, and future directions

This study offers several notable strengths, reinforcing the validity of its findings. First, its prospective design allows for real-time data collection, minimizing recall bias. Additionally, consistent follow-up until patient discharge ensures reliable assessment of postoperative pain, motor function, and opioid consumption. The homogeneity in surgical technique across all groups further strengthens the study by reducing variability and procedural bias. Notably, the standardized DSB protocol using fixed volumes and concentrations at two precise anatomical locations enhanced reproducibility. Patients received rescue opioids only as needed, allowing clear comparisons in opioid consumption across techniques. Additionally, the study was adequately powered (90%) with 40 participants per group, minimizing Type II error.

Despite these strengths, this study has limitations. While conducted at a single center with a modest sample size, its findings warrant validation in larger, multicenter trials. Moreover, DSB has been assessed only in primary TKA via a medial approach. Its applicability in revision surgeries or alternate surgical techniques remains unexplored. Long-term pain outcomes were beyond the scope of this study and should be evaluated in future research. These directions will determine DSB’s full clinical potential across diverse TKA contexts.

Data availability

The data that support the findings of this study are openly available in the Zenodo repository [47].

## Conclusions

In conclusion, DSB marks a paradigm shift in RA for TKA, offering comprehensive, procedure-specific analgesia with superior opioid-sparing and motor-sparing benefits and patient satisfaction, fully aligned with ERAS principles. Its consistent coverage, elimination of rescue opioid use, and enhanced functional outcomes position it as a strong contender to replace traditional single-shot techniques. Given these advantages, adopting DSB as a standard analgesic technique in TKA is justified and timely. With further refinement and broader validation, DSB is poised to become the new effective RA option in TKA pain management.
